# Trends in U.S. Emergency Department Visits Related to Suspected or Confirmed Child Abuse and Neglect Among Children and Adolescents Aged <18 Years Before and During the COVID-19 Pandemic — United States, January 2019–September 2020

**DOI:** 10.15585/mmwr.mm6949a1

**Published:** 2020-12-11

**Authors:** Elizabeth Swedo, Nimi Idaikkadar, Ruth Leemis, Taylor Dias, Lakshmi Radhakrishnan, Zachary Stein, May Chen, Nickolas Agathis, Kristin Holland

**Affiliations:** ^1^Community Interventions and Critical Populations Task Force, CDC COVID-19 Response Team; ^2^Division of Injury Prevention, National Center for Injury Prevention and Control, CDC; ^3^Division of Violence Prevention, National Center for Injury Prevention and Control, CDC; ^4^Division of Health Informatics and Surveillance, Center for Surveillance, Epidemiology, and Laboratory Services, CDC; ^5^Deloitte, New York, New York; ^6^ICF, Atlanta, Georgia; ^7^Epidemic Intelligence Service, CDC; ^8^Division of Overdose Prevention, National Center for Injury Prevention and Control, CDC.

Heightened stress, school closures, loss of income, and social isolation resulting from the coronavirus disease 2019 (COVID-19) pandemic have increased the risk for child abuse and neglect (*1*). Using National Syndromic Surveillance Program (NSSP) data from January 6, 2019–September 6, 2020, CDC tabulated weekly numbers of emergency department (ED) visits related to child abuse and neglect and calculated the proportions of such visits per 100,000 ED visits, as well as the percentage of suspected or confirmed ED visits related to child abuse and neglect ending in hospitalization, overall and stratified by age group (0–4, 5–11, and 12–17 years). The total number of ED visits related to child abuse and neglect began decreasing below the corresponding 2019 period during week 11 (March 15–March 22, 2020) for all age groups examined, coinciding with the declaration of a national emergency on March 13 (*2*); simultaneously, the proportion of these visits per 100,000 ED visits began increasing above the 2019 baseline for all age groups. Despite decreases in the weekly number of ED visits related to child abuse and neglect, the weekly number of these visits resulting in hospitalization remained stable in 2020; however, the yearly percentage of ED visits related to child abuse and neglect resulting in hospitalization increased significantly among all age groups. Although the increased proportion of ED visits related to child abuse and neglect might be associated with a decrease in the overall number of ED visits, these findings also suggest that health care–seeking patterns have shifted during the pandemic. Hospitalizations for child abuse and neglect did not decrease in 2020, suggesting that injury severity did not decrease during the pandemic, despite decreased ED visits. Child abuse is preventable; implementation of strategies including strengthening household economic supports and creating family-friendly work policies can reduce stress during difficult times and increase children’s opportunities to thrive in safe, stable, and nurturing relationships and environments (*3*).

Despite known risk for child abuse and neglect during pandemics (*4*) and preliminary reports of increased severity of child abuse and neglect in some facilities (*5*), official reports to child protection agencies have declined across the United States by 20%–70%, attributed to decreased in-person contact between children and mandated reporters (e.g., teachers, social workers, and physicians) (*6*). Lack of timely data on child abuse and neglect in the context of COVID-19 highlights the value of near real-time data from NSSP, which provide the opportunity to examine trends in ED visits and hospitalizations for suspected or confirmed child abuse and neglect before and during the COVID-19 pandemic.

Data for U.S. ED visits among children and adolescents aged <18 years were obtained from NSSP’s BioSense Platform using a query for suspected and confirmed ED visits related to child abuse and neglect developed by NSSP, CDC’s Division of Violence Prevention, and local and state health departments (*7*). NSSP is a collaboration among CDC, federal partners, local and state health departments, and academic and private sector partners to support the collection and analysis of electronic health data from EDs, urgent and ambulatory care centers, inpatient health care facilities, and laboratories. As of March 31, 2020, a total of 3,310 EDs in 47 states and the District of Columbia contributed data to the platform daily, providing information on approximately 73% of all ED visits in the United States. Visits were included if the ED provider or facility documented suspected or confirmed physical, sexual, or emotional abuse or physical or emotional neglect of a child or adolescent aged <18 years by a parent or other caregiver (*8*). To limit the impact of data quality on resulting trends, only visits from facilities that consistently sent informative* discharge diagnoses for ≥70% of cases with ≤20% standard deviation were included; the number of facilities meeting these criteria varied from week to week but averaged 2,970 facilities during the study period (approximately 90% of NSSP’s participating EDs).

Data were analyzed to examine national trends in ED visits for suspected or confirmed child abuse and neglect during January 6, 2019–September 6, 2020, the period before and during the U.S. COVID-19 pandemic. Weekly numbers and proportions of visits related to child abuse and neglect per 100,000 ED visits were computed overall and stratified by age group (0–4, 5–11, and 12–17 years). In addition, weekly and annual^†^ percentages of ED visits related to child abuse and neglect resulting in hospitalization were calculated. The change in mean ED visits related to child abuse and neglect per week during the early pandemic period (March 31–April 27, 2020) and the comparison period (March 29–April 25, 2019) was calculated as the mean difference in total ED visits related to child abuse and neglect between the two 4-week periods. Statistically significant differences in annual percentages of ED visits related to child abuse and neglect ending in hospitalizations were assessed using t-tests. All analyses were performed using R software (version 4.0.2; The R Foundation). This activity was reviewed by CDC and was conducted consistent with applicable federal law and CDC policy.^§^

The total number of 2020 ED visits meeting the syndrome definition for child abuse and neglect (Table) began decreasing to below the number of visits that occurred during the corresponding 2019 prepandemic period in week 11 (March 15–March 22), coinciding with the president’s Proclamation Declaring a National Emergency Concerning the Novel Coronavirus Disease (COVID-19) Outbreak on March 13, 2020 (Figure 1). This pattern was observed for all age groups examined (Supplementary Figure, https://stacks.cdc.gov/view/cdc/98213). At the same time, the proportion of ED visits related to child abuse and neglect per 100,000 ED visits began increasing above the proportion seen during the corresponding period in 2019 (Figure 1). ED visits related to child abuse and neglect among children and adolescents aged <18 years reached their nadir during week 13 (March 29–April 4, 2020). During the 4-week period following this early pandemic nadir (March 29–April 25), the number of ED visits related to child abuse and neglect among children and adolescents aged <18 years averaged 53% less than the number that occurred during the corresponding period in 2019 (March 31–April 27) (Figure 1). The number of ED visits related to child abuse and neglect was lower during this period in 2020, compared with visits during the corresponding period in 2019 for every age group, with the largest proportional declines in number of visits by children aged 5–11 years (61%) (Supplementary Figure, https://stacks.cdc.gov/view/cdc/98213).

**TABLE:** Syndrome definition description and chief complaint search terms, diagnosis codes, and negations included in syndrome definitions for emergency department visits related to suspected and confirmed child abuse and neglect — United States, January 2019–September 2020

Outcome	Description of syndrome definition	Chief complaint search terms*	Diagnosis codes	Negations^†^
Suspected and confirmed child abuse and neglect	The suspected and confirmed child abuse and neglect (CAN) syndromic surveillance definition uses *International Classification of Diseases, Ninth Revision, Clinical Modification* (ICD-9-CM) codes *International Classification of Diseases, Tenth Revision, Clinical Modification* (ICD-10-CM) codes, Systematized Nomenclature of Medicine (SNOMED) codes, and free text terms to detect cases of suspected child abuse and neglect in emergency department and ambulatory health care settings. For the purposes of the CAN syndromic definition, suspected child abuse or neglect visits are categorized as visits related to suspected or confirmed physical, sexual, or emotional abuse; or physical or emotional neglect as perpetrated by parents, caregivers, or an authorized custodian of the child. Acts of violence perpetrated by peers, siblings, or intimate partners are excluded from the CAN definition.	Sexual Abuse Nurse Exam (SANE),^§^ non-accidental trauma (NAT), neglect, abuse, Child to Adult Abuse Response Team (CAART), abandon, forensic,^§^ molest, Forensic Nurse Exam (FNE),^§^ rape,^§^ assault,^§^ Sexual Assault Forensic Examiner (SAFE),^§^ Sexual Abuse Response Team (SART),^§^ force sex,^§^ suspected sexual,^§^ alleged sexual* ^§^AND mother, mom, stepmom, grandmom, fostermom, grandma, grandpa, stepdad, fosterdad, granddad, babysitter, nanny, parent, fosterparent, stepparent, grandparent, custodian, guardian, uncle, aunt	T74.02, T74.12, T74.22, T74.32, T74.4, T74.52, T74.62, T74.72, T74.92, T76.02, T76.12, T76.22, T76.32, T76.4, T76.52, T76.62, T76.72, T76.92, Z04.81, Z04.82, Z04.42,^¶^ Z04.72,^¶^ Y07.1, Y07.4, Y07.5, Y07.6, Y07.9, 995.5, E904.0, E967.1, E967.2, E967.6, E967.7, E967.8, E967.9, V71.5, V71.81, 432464008, 713834002, 777996001, 418189009, 386702006, 242037000, 162596006, 397940009, 702954001, 242037000, 95930005, 397660003, 217635005, 217634009, 217633003, 697951004, 12399131000119105, 473453008, 371775004, 700254002, 95922009, 228143000, 697949003, 12242871000119109, 371779005, 397864009, 237461000119103, 700229002, 720824009, 225824003, 720823003, 225826001, 371776003, 397660003, 777996001, 102458000, 213015009, 419261006, 430139008, 225823009, 361217003, 713821003, 41358001, 23776007, 70167006, 51347003, 213017001, 242046006, 361217003	Y07.0 Y07.4 E967.3 E967.4 E967.5 433960002 Athero Left side or right side Stroke Medical neglect Alcohol Drug Substance Polysubstance Cannabis Weed Hallucinogen THC Marijuana History of abuse Xanax Stimulant Meth Cocaine Tobacco Opioid Inhalant Denies any abuse House Mill Building Motor vehicle Intoxication DUI Crash Molestacion Molestia Molestar Molesta Molesto Molestra Molestio Assaulted by friend Assaulted by boyfriend Assaulted by girlfriend Assaulted by sibling Assaulted by brother Assaulted by sister Assaulted by half brother Assaulted by half sister Assaulted by classmate Denies assault Enforcement Urin No force Sexton Air force Chemo

* To maintain specificity of the query, certain chief complaint terms were included in the search only when paired with parent/caregiver perpetrator terms to exclude potential cases of peer or intimate partner violence.

^†^ Coding very specific negations can be used to negate false positives.

^§^ These chief complaint terms are only included when paired with a parent/caregiver perpetrator term, such as mother, mom, stepmom, grandmom, fostermom, grandma, grandpa, stepdad, fosterdad, granddad, babysitter, nanny, parent, fosterparent, stepparent, grandparent, custodian, guardian, uncle, aunt.

^¶^ ICD-10-CM codes Z04.42 (“Suspected child sexual abuse, ruled out”) and Z04.72 (“Suspected physical abuse, ruled out”) were included in the CAN query because, during query validation, this code was found to be inconsistently applied to both identify and rule out cases.

**FIGURE 1:**
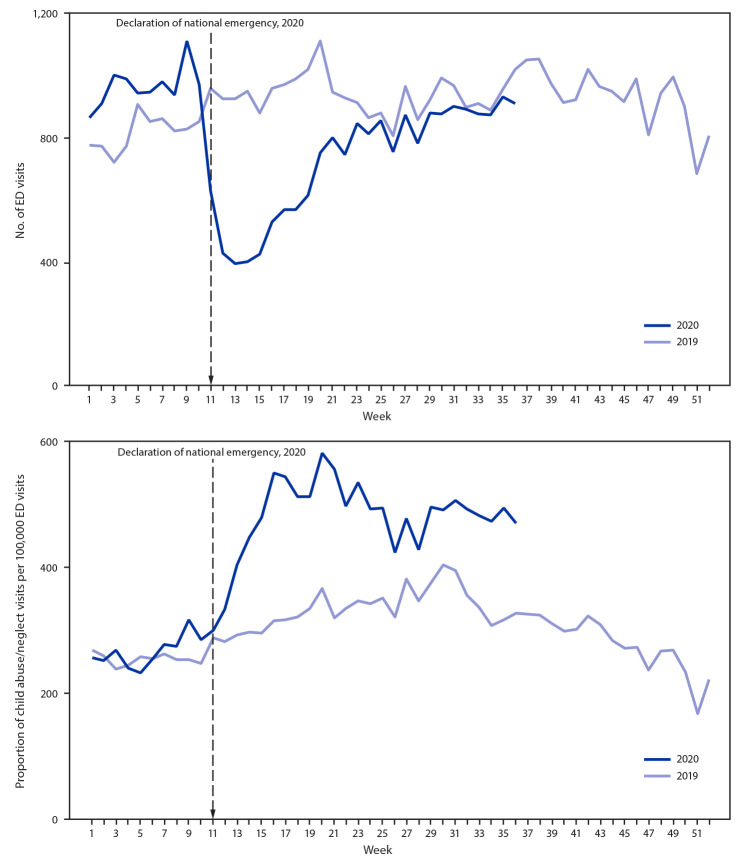
Number (A) and proportion (B) of emergency department (ED) visits related to suspected and confirmed child abuse and neglect among children and adolescents aged <18 years, by week — National Syndromic Surveillance Program, United States, 2019–2020

Despite decreases in the total number of ED visits related to child abuse and neglect, the number of these ED visits resulting in hospitalization did not decline in 2020 (Figure 2). As a result of the consistent number of hospitalizations and the decrease in the number of overall ED visits, the percentage of ED visits related to child abuse and neglect ending in hospitalization increased significantly among children and adolescents aged <18 years, from 2.1% in 2019 to 3.2% in 2020 (p<0.001) (Figure 2). Significant increases in the percentage of ED visits related to child abuse and neglect ending in hospitalization were also observed for children aged 0–4 years (3.5% in 2019 versus 5.3% in 2020; p<0.001) and 5–11 years (0.7% in 2019 versus 1.3% in 2020; p<0.001), and adolescents aged 12–17 years (1.6% in 2019 versus 2.2% in 2020; p = 0.002) (Supplementary Figure, https://stacks.cdc.gov/view/cdc/98213).

**FIGURE 2:**
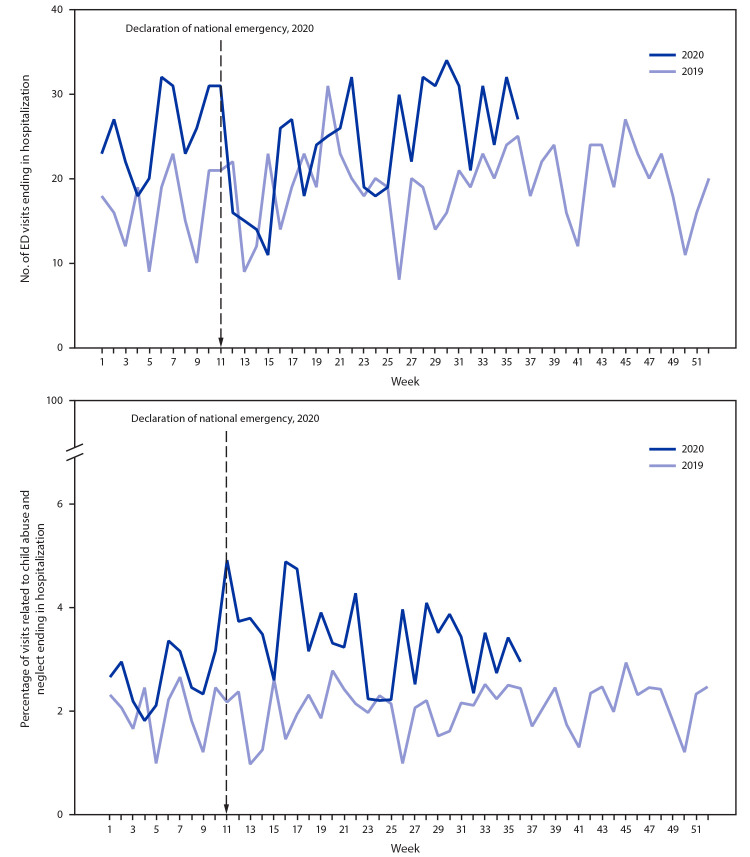
Number (A) and percentage (B) of emergency department (ED) visits related to suspected and confirmed child abuse and neglect ending in hospitalization among children and adolescents aged <18 years, by week — National Syndromic Surveillance Program, United States, 2019–2020

## Discussion

ED visits related to suspected or confirmed child abuse and neglect decreased beginning the week of March 15, 2020, coinciding with the declaration of a national emergency related to COVID-19 and implementation of community mitigation measures (*5*). The 53% decrease in ED visits related to child abuse and neglect among children aged <18 years in early 2020 compared with the number of visits during early 2019 mirrors trends reported for all ED visits; during weeks 13–16 of 2020, the volume of U.S. ED visits declined by 72% among children aged ≤10 years and 71% among children and adolescents aged 11–14 years compared with ED visits during 2019 (*9*). Although the total number of ED visits related to child abuse and neglect decreased, the proportion of these visits per 100,000 ED visits increased, suggesting that health care–seeking patterns shifted during the pandemic, with ED visits for other causes declining more than ED visits for child abuse and neglect declined. Despite the ongoing pandemic, caregivers were more likely to take children to EDs for evaluation of complaints related to child abuse and neglect relative to other chief complaints. This pattern might reflect decreased health care–seeking for other medical complaints or a need to seek medical care because of persistence or worsening of child abuse and neglect. The decreased number of ED visits related to child abuse and neglect coincides with decreases in reports of child abuse and neglect to child protective services (*4*). The consistent number of visits related to child abuse and neglect requiring hospitalization from 2019 to 2020, despite decreased number of ED visits related to child abuse and neglect, suggests that injury severity did not decrease during the pandemic.

The COVID-19 pandemic and the social and economic effects of mitigation measures, such as loss of income, increased stress related to parental child care and schooling responsibilities, and increased substance use and mental health conditions among adults (*10*), increase the risk for child abuse and neglect. These pandemic-related risk factors might be tied to the observed increased proportions of ED visits related to child abuse and neglect.

The findings in this report are subject to at least six limitations. First, the denominator for proportion estimates declined substantially during the pandemic, making interpretation of temporal proportion trends more difficult. Second, the number of facilities participating in NSSP might change over time, as facilities are added, and, more rarely, as they close. Proportions and counts might be influenced by characteristics of the populations served by participating facilities. Third, the syndrome definition used in this analysis might under- or overestimate facility visits related to suspected or confirmed child abuse and neglect because of jurisdictional or temporal differences in coding, reporting, or availability of chief complaints and discharge diagnoses. To minimize the impact of fluctuating data quality, only data from the most consistently reporting facilities were used. Fourth, NSSP data are not nationally or regionally representative, and results are not generalizable to nonparticipating facilities. Fifth, the data source does not distinguish between incident and recurrent health care facility visits; thus, interpretation of results is limited to ED visits, not patients. Finally, data were transmitted to NSSP in near real-time and are not considered final; results might change over time as additional data are added.

Continued surveillance of child abuse and neglect during the pandemic is warranted, and syndromic surveillance data enable the monitoring of these outcomes in near real-time. Importantly, this report demonstrates that ED visits related to abuse and neglect declined during the COVID-19 pandemic, despite evidence that pandemics increase risk for child abuse and neglect (*1*). Identification and support of alternative means to detect and report child abuse and neglect is needed during the COVID-19 pandemic. Because of the numerous negative consequences of child abuse and neglect on children’s short-term and long-term physical and mental health (*6*), further research into the epidemiology of child abuse and neglect during the COVID-19 pandemic (e.g., risk factors and protective factors, types of abuse observed, types of injuries sustained, and reasons for hospitalization) is needed to better understand the pandemic’s effects on child abuse and neglect.

Child abuse and neglect is preventable. CDC’s technical package for preventing child abuse and neglect outlines prevention strategies based on the best available evidence, some of which might be particularly useful during public health emergencies (*6*). These prevention opportunities include strengthening families’ economic supports, ensuring family-friendly work policies so that parents can continue to work while balancing childcare responsibilities, and modifying early home visitation practices to be virtual while social distancing measures are in effect. Broad implementation of prevention strategies can reduce child abuse and neglect and help ensure that children and adolescents experience safe, stable, nurturing relationships and environments (*6*).

**Summary**

**What is already known about this topic?**

Public health emergencies increase risk for child abuse and neglect because of increased stressors and loss of financial and social supports.

**What is added by this report?**

During the COVID-19 pandemic, the total number of emergency department visits related to child abuse and neglect decreased, but the percentage of such visits resulting in hospitalization increased, compared with 2019.

**What are the implications for public health practice?**

The pandemic has affected health care–seeking patterns for child abuse and neglect, raising concerns that victims might not have received care and that severity of injuries remained stable or worsened. Implementation of strategies to prevent child abuse and neglect is important, particularly during public health emergencies.

## Footnotes

* Discharge diagnoses were not null or did not simply include terms like “unknown.”

† Complete year for 2019 compared with partial year for 2020.

§ 45 C.F.R. part 46.102(l)(2), 21 C.F.R. part 56; 42 U.S.C. Sect. 241(d); 5 U.S.C. Sect. 552a; 44 U.S.C. Sect. 3501 et seq.
