# *Erratum:* Vol. 69, No. 49

**DOI:** 10.15585/mmwr.mm7002a4

**Published:** 2021-01-15

In the report “Trends in U.S. Emergency Department Visits Related to Suspected or Confirmed Child Abuse and Neglect Among Children and Adolescents Aged <18 Years Before and During the COVID-19 Pandemic — United States, January 2019-September 2020,” on page 1842, the fourth sentence in the third complete paragraph should have read “The change in mean ED visits related to child abuse and neglect per week during the early pandemic period (**March 29–April 25, 2020**) and the comparison period (**March 31–April 27, 2019**) was calculated as the mean difference in total ED visits related to child abuse and neglect between the two 4-week periods.”

